# The Association Between Mild Cognitive Impairment and Physical Function in Older Japanese Adults Aged 75 Years or Older Living in Independent Senior Housing: A Cross-Sectional Study

**DOI:** 10.3390/healthcare12212106

**Published:** 2024-10-23

**Authors:** Kanako Ohno, Shuji Sawada, Naho Fujimaki, Kyoko Sakai, Sawako Wakui, Nobuto Shibata, Nobuhiro Sato, Hisashi Naito, Shuichi Machida

**Affiliations:** 1 Graduate School of Health and Sports Science, Juntendo University, Chiba 270-1695, Japan; kanako-ono@e-life-design.co.jp (K.O.); hnaitou@juntendo.ac.jp (H.N.); 2 Tokyu E-Life Design Co., Ltd., Tokyo 150-0043, Japan; naho-fujimaki@e-life-design.co.jp (N.F.); kyoko-sakai@e-life-design.co.jp (K.S.); 3 Faculty of Health and Sports Science, Juntendo University, Chiba 270-1695, Japan; sh-sawada@juntendo.ac.jp (S.S.); swakui@juntendo.ac.jp (S.W.); 4 Juntendo Tokyo Koto Geriatric Medical Center, Tokyo 136-0075, Japan; nshibata@juntendo.ac.jp; 5 Graduate School of Medicine, Juntendo University, Tokyo 113-8421, Japan; nsato@juntendo.ac.jp

**Keywords:** cognitive function, gait speed, Montreal Cognitive Assessment, muscle strength

## Abstract

**Background/Objective**: Although there are many reports on the association between cognitive and physical functions in older adults, little information is available on those aged ≥75 years. Therefore, this study aimed to determine whether mild cognitive impairment (MCI) in older adults over 75 years who live in independent senior housing is associated with physical function. **Methods**: In this study, 271 participants (174 women and 97 men) with a mean age of 85.4 ± 4.7 years were included. Cognitive function was assessed using the Japanese version of the Montreal Cognitive Assessment; a score < 26 confirmed MCI. MCI was an objective variable in univariate and multivariable logistic regression analyses. Physical function was measured using hand grip strength, normal and maximum gait speeds, and the 30 s chair stand test. Physical function was an explanatory variable adjusted for age and divided into tertiles (high, middle, and low) based on sex. The significance level was set at 5%. **Results**: There were 170 participants (63%) with MCI. Compared to the non-MCI group, the MCI group had significantly higher age and significantly lower normal and maximum gait speeds and 30 s chair stand test values Age-adjusted univariate analyses in women showed higher MCI rates in the low-fitness group than in the high-fitness group for maximum gait speed and 30 s chair stand test values. No variables were associated with MCI in men. **Conclusions**: MCI may be associated with physical function in women and older adults over 75 years who live in independent senior housing.

## 1. Introduction

Dementia is a progressive neurocognitive disorder characterized by cognitive decline [1], which has become a major public health problem with an increasing number of affected individuals worldwide. Mild cognitive impairment (MCI) is a clinical condition that is intermediate between normal cognitive aging and dementia [2] and often precedes dementia. In older individuals, MCI is associated with a high risk of progression to dementia. A study reported that older adults with MCI develop Alzheimer’s disease at a rate of 10–30% annually, compared with that of 1–2% annually in older adults with non-MCI [3]. Furthermore, several studies have suggested that the incidence, prevalence, severity, and progression of cognitive impairment are affected by sex differences [4,5].

Cognitive and physical functions are reportedly associated with older age. A comfortable walking speed is associated with comprehensive cognitive impairment in community-dwelling older adults [6] and older adults receiving primary care services [7]. A study measuring various physical functions such as grip strength, gait speed, and chair stand among community-dwelling older adults, with a mean age of 72.5 ± 5.2 years, reported that the cognitively impaired group had significantly poorer physical function than the normal group [8]. Investigating and characterizing the association between MCI and physical function may aid in the early detection and prevention of its transition to dementia.

To date, many studies have investigated the association between cognitive and physical functions in older individuals, although most of these studies have focused on older people with an average age in the 70s [7,9,10,11,12]. Japan has the highest proportion of older people in the population than any other country in the world. In Japan, older people are generally defined as those aged 65 or older, and the latter-stage elderly are those aged 75 or older, in whom both cognitive and physical functions are expected to decline rapidly.

Senior housing has standardized entrance fees, monthly fees, room sizes, building structures, etc., and residents are a group of people with roughly homogeneous living environments, with socioeconomic factors controlled to a certain extent. The number of users of senior housing in Japan and worldwide is increasing. Although there are many studies that focus on community-dwelling older people, there are few studies that focus on residents of independent senior housing. It is possible that results may differ between studies targeting community-dwelling older people and studies targeting residents of independent senior housing. Therefore, the purpose of this study was to investigate the association between mild cognitive impairment and physical function in older adults over 75 years who live in independent senior housing.

## 2. Materials and Methods

### 2.1. Participants

A total of 324 residents (214 women, 110 men, mean age 85.2 ± 5.3 years) participated in physical fitness tests conducted at 12 independent senior housing facilities, of which 313 people aged 75 years or older were the participants of this study. This study was conducted in accordance with and conforms to the provisions of the Declaration of Helsinki (as revised in Brazil 2013). This study was approved by the Ethics Committee of Juntendo University (Approval Number: 2021–81), and written informed consent was obtained from all participants.

### 2.2. Physical Function Measures

The following parameters were measured at the annual physical fitness test. Hand grip strength (kg) was measured by using a Smedley hand dynamometer (Grip-D T.K.K. 5401, Takei Instruments Co., Ltd., Niigata, Japan). The measurements were performed twice on the right and left hands. The highest values of four measurements were used in the analysis. The measurements were performed according to the methods recommended in the consensus report on the assessment of sarcopenia in Asia [13].

The gait speed was measured during normal and maximal walking movements. Walking paths were 7 m or 12 m, and walking time (s) was measured for the middle 5 m or 10 m. Differences in walkway length have been shown to have no effect on either comfortable or maximum walking speed [14]. Walking time was measured using a digital stopwatch (HS-3C-8AJH, CASIO COMPUTER CO., LTD., Tokyo, Japan). Gait speed (m/s) was calculated by dividing the walking distance (m) by the walking time (s). Measurements were obtained twice for each participant, and the faster value was used for analysis.

For the 30 s chair stand test (CS-30), the participants performed a 30 s sit-to-stand trial [15]. The participants were instructed to sit on the front edge of a 40 cm high seat with their arms crossed in front of their chest. Furthermore, they were instructed to stand up (to maximum knee extension, based on body sense) and sit down as quickly as possible for 30 s. The number of times the buttocks touched the seat surface was recorded. Time (30 s) was measured using a digital stopwatch (HS-3C-8AJH, CASIO COMPUTER CO., LTD., Tokyo, Japan). The CS-30 test can be used to evaluate lower-extremity muscle strength in older Japanese adults [16], and our previous study reported that the test–retest reliability using the ICC was 0.78 [17].

### 2.3. Cognitive Function Measures

Cognitive function was assessed using the Japanese version of the Montreal Cognitive Assessment (MoCA-J), which assesses global cognition, including visuospatial and executive functions, naming, attention, language, abstraction, delayed recall, and orientation.

MoCA-J scores range from 0 to 30, with higher scores indicating better cognitive function, and a score of <26 indicating MCI [18].

### 2.4. Other Measurements

Information regarding age and sex was collected using a questionnaire. Body weight (kg) and skeletal muscle mass (kg) were measured using InBody 470 (InBody Japan Inc. Tokyo, Japan), which uses bioelectrical impedance analysis to estimate body composition. Body mass index (BMI) was calculated by dividing the body weight (kg) by height squared (m^2^). The skeletal muscle index (SMI) was calculated by dividing appendicular skeletal muscle mass (kg) by height squared (m^2^).

### 2.5. Statistical Analyses

Data are presented as the mean ± standard deviation for continuous variables and number (%) for categorical variables. Based on the MoCA-J score, participants were classified into MCI (MoCA-J score < 26) and non-MCI (MoCA-J score ≥ 26) groups.

For group comparisons of continuous variables, normality tests were first performed using the Kolmogorov–Smirnov test. Student’s unpaired *t*-test was used for group comparisons of continuous variables showing a normal distribution. For continuous variables that did not show a normal distribution, the Mann–Whitney U test was used. The chi-squared test was used for group comparisons of categorical variables.

The presence of MCI was used as the objective variable, and the explanatory variables were age, hand grip strength, normal gait speed, maximum gait speed, and CS-30. Univariate and multivariable logistic regression analyses were used to estimate the odds ratio (OR) and 95% confidence interval (CI) for each. Age was divided into two groups (≤84 years and ≥85 years), and physical function (hand grip strength, normal gait speed, maximum gait speed, and the CS-30) was divided into three groups (high, middle, and low) in tertiles by sex, respectively. Additionally, we adjusted for age, estimated OR, and 95% CI.

The level of significance was set at *p* < 0.05. All statistical analyses were performed using SPSS version 27 (IBM, Armonk, NY, USA).

## 3. Results

A total of 313 individuals participated in this study; 42 participants who did not complete physical or cognitive measures were excluded. Hence, the final study included 271 participants. Table 1 presents the characteristics of the participants, among whom 174 (64.2%) were women, and the average age was 85.4 ± 4.7 years. There were 170 (62.7%) participants with MCI and 101 (32.3%) with non-MCI. The MoCA-J scores for the non-MCI and MCI groups were 27.4 ± 1.2 and 21.9 ± 3.0, respectively (*p* < 0.001).

Table 2 shows the characteristics of the non-MCI and MCI participants for women and men, respectively.

**Figure 1:**
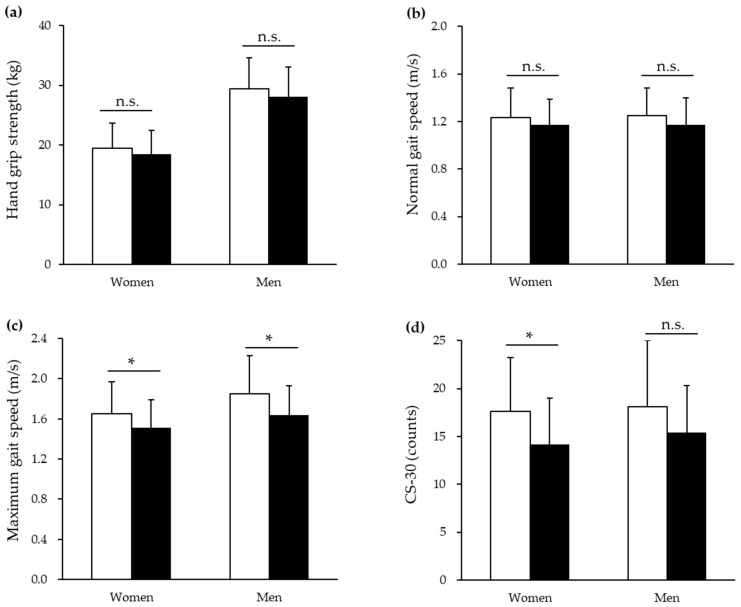
Physical functions of non-MCI and MCI participants for women and men. Abbreviations: □: non-MCI group; ■: MCI group; MCI, mild cognitive impairment; CS-30, 30 s chair stand. Values are means ± standard division. * *p* < 0.05. n.s.: not significant.

Figure 1 shows the physical functions of non-MCI and MCI participants for women and men, respectively. In women, hand grip strength was 19.5 ± 4.2 in the non-MCI group and 18.5 ± 3.9 in the MCI group; normal gait speed was 1.23 ± 0.25 in the non-MCI group and 1.17 ± 0.22 in the MCI group; maximum gait speed was 1.65 ± 0.32 in the non-MCI group and 1.51 ± 0.28 in the MCI group; and CS-30 was 17.6 ± 5.6 in the non-MCI group and 14.2 ± 4.8 in the MCI group. Significant differences were observed between the non-MCI and MCI groups for maximum gait speed and CS-30. In men, hand grip strength was 29.4 ± 5.2 in the non-MCI group and 28.1 ± 5.0 in the MCI group; normal gait speed was 1.25 ± 0.23 in the non-MCI group and 1.17 ± 0.23 in the MCI group; maximum gait speed was 1.85 ± 0.38 in the non-MCI group and 1.64 ± 0.29 in the MCI group; and CS-30 was 18.1 ± 6.9 in the non-MCI group and 15.4 ± 4.9 in the MCI group. Significant differences were observed between the non-MCI and MCI groups for maximum gait speed.

Table 3 reveals that the univariate analysis in women showed a higher incidence of MCI in the low-fitness group than in the high-fitness group for grip strength, maximal gait speed, and CS-30 values. Age-adjusted univariate analyses in women showed higher rates of MCI in the low-fitness group than in the high-fitness group for maximal gait speed and CS-30 values.

In contrast, the univariate analysis in men showed a higher incidence of MCI in the low-fitness group than in the high-fitness group in terms of maximal gait speed. However, there were no variables associated with MCI for the age-adjusted univariate analysis in men (Table 4).

## 4. Discussion

This study investigated the association between mild cognitive impairment and physical function in older adults over 75 years who live in independent senior housing. Maximal gait speed and CS-30 were associated with a higher incidence of MCI in the low-fitness group than in the high-fitness group among older women, with CS-30 exhibiting the strongest association. Among older men, no variables were associated with MCI.

It has been reported that a decline in gait performance is associated with the onset of dementia [19,20,21]. Previous studies have shown an association between cognitive function and usual gait speed in older adults [6,9,22,23,24]. However, this study found no association between usual gait speed and cognitive function. We believe that the reason for the different results is due to differences in the average usual gait speed. The mean usual gait speed of participants in the study of older people using primary care services and older people living in the community was 0.9 m/s [6] and 0.95 m/s [9], respectively, but the mean usual gait speed of participants in the current study was 1.20 m/s for women and 1.19 m/s for men, which was faster than the participants in the previous study. The standard for normal gait speed is 1.0 m/s [25], and in a previous study, participants who walked at less than 1.0 m/s had a decline in gait ability, but the participants in this study maintained their gait ability. Although the average age of the participants in this study was older than the subjects in the previous study, their gait ability was higher, which may have influenced the difference in results. A study investigating the relationship between self-paced gait speed and general cognitive function in older adults with a mean age of 89.4 years also found a positive correlation between gait speed and cognitive function [26]. The mean gait speed of the participants in the above study was 0.45 m/s, and 31% used walking aids, so differences in gait ability compared to the participants in this study may have influenced the results. Previous studies have also shown an association between cognitive function and maximum gait speed in older adults [7,9,10,11]. It has been reported that maximum gait speed is more closely related to cognitive function than normal gait speed because it requires effort to walk at a fast pace [9]. In this study, maximum gait speed in women was associated with cognitive function. On the other hand, no association was found between maximum gait speed and cognitive function in men. The average maximum gait speed was significantly higher in men than in women. In addition, the average maximum gait speed in previous studies where an association was found was often lower than the average value for men in this study, and the average age was also more than 10 years younger, so it is possible that the maximum gait speed value of the men in this study was higher and their physical function level was higher. Therefore, it is possible that for the men with a higher level of physical function, it was not associated with cognitive function.

This study showed a significant association between CS-30 and MCI in women. However, no significant association was found between CS-30 and MCI in men. A study comparing CS-30 scores between MCI and non-MCI groups in community-dwelling older adults found no significant difference [12]. Furthermore, it has been reported that CS-30 scores and Mini Mental State Examination (MMSE) scores were not significantly correlated [7], which differs from the results of this study. However, these studies mixed men and women in their analyses. Another study analyzed the relationship between CS-30 scores and cognitive function separately in men and women, as in this study [27]. In women, the group with low lower-limb function had significantly lower cognitive function than the group with high lower-limb function, but no significant difference was found in men, which is consistent with the results of this study. The participants in the study in which there was no significant difference in CS-30 were in their early 70s, whereas participants in the study in which an association between cognitive function and CS-30 was found only in women were in their late 70s [27]. Because the participants in this study were in their 80s, the association between cognitive function and CS-30 may be related to age as well as gender. This may be due to the fact that women have weaker muscle strength than men. In this study, CS-30 showed the strongest association with the incidence of MCI in women. Previous studies have shown a significant correlation between CS-30 scores and knee extension strength per body weight [16], and CS-30 is used as an assessment of leg muscle strength. It is suggested that leg muscle strength may be the physical function most strongly associated with MCI in women aged 75 years or older living in independent senior housing.

The cross-sectional nature of this study limits the ability to draw causal inferences between mild cognitive impairment (MCI) and physical function. Therefore, it is necessary to verify these causal relationships using longitudinal data in the near future. Recent studies [28,29] have demonstrated the effectiveness of resistance training at improving cognitive function. This study’s results will provide useful data when considering measures to prevent cognitive decline among older adults, particularly through interventions targeting physical function and muscle strength.

Our study had some limitations. First, although education, several diseases, socioeconomic status, and exercise habits have been reported to affect cognitive function, we did not obtain these data. We did not consider other types of cognitive impairment that may be present in the sample other than mild cognitive impairment (e.g., dementia), which limits the scope of our results, and the results may not apply to individuals with more severe cognitive impairment. Second, the sample size of men and that of the reference group was small when logistic regression analysis was conducted. This disparity between male and female participants may affect the robustness of the results, especially when making claims about gender differences. Third, this was a cross-sectional study, which means that the causal association between cognitive and physical functions could not be determined. Fourth, the study focused on residents of independent senior housing and may not be representative of the broader population, such as seniors living in the community. Hence, further studies are needed to overcome these limitations.

## 5. Conclusions

In conclusion, mild cognitive impairment may be associated with physical function in women and in older adults over 75 years who live in independent senior housing, and the CS-30 may be the tool most strongly associated with MCI in women.

## Figures and Tables

**Table 1:** Characteristics of participants.

	All		Women		Men		
	(n = 271)		(n = 174)		(n = 97)		
Age (years)	85.4 ± 4.7	(75–100)	85.0 ± 4.8	(75–97)	86.1 ± 4.4	(76–100)	^b^
Height (cm)	154.6 ± 8.5	(134.6–178.3)	149.7 ± 5.6	(134.6–164.5)	163.2 ± 5.4	(150.5–178.3)	^b^*
Weight (kg)	52.4 ± 9.4	(31.1–77.6)	48.1 ± 7.6	(31.1–69.2)	59.9 ± 7.2	(38.4–77.6)	^a^*
BMI (kg/m^2^)	21.9 ± 3.1	(13.6–29.6)	21.5 ± 3.3	(13.6–29.6)	22.5 ± 2.4	(14.8–29.4)	^b^*
SMI (kg/m^2^)	6.08 ± 0.96	(3.86–9.17)	5.58 ± 0.63	(3.86–7.06)	7.02 ± 0.74	(5.23–9.17)	^b^*
MoCA-J score (points)	24.0 ± 3.6	(12–30)	24.1 ± 3.7	(13–30)	23.7 ± 3.4	(12–30)	^b^
MCI, n (%)	170 (62.7%)		101 (58.0%)		69 (67.6%)		^c^*
Hand grip strength (kg)	22.4 ± 6.4	(6.3–46.1)	19.0 ± 4.0	(6.3–28.7)	28.5 ± 5.1	(16.8–46.1)	^b^*
Normal gait speed (m/s)	1.20 ± 0.24	(0.54–1.79)	1.20 ± 0.24	(0.60–1.68)	1.19 ± 0.24	(0.54–1.79)	^a^
Maximum gait speed (m/s)	1.62 ± 0.32	(0.78–2.54)	1.57 ± 0.30	(0.87–2.31)	1.70 ± 0.33	(0.78–2.54)	^a^*
CS-30 (counts)	15.8 ± 5.5	(5–34)	15.6 ± 5.4	(5–34)	16.1 ± 5.6	(5–32)	^b^

Abbreviations: BMI, body mass index; SMI, skeletal muscle index; MoCA-J, the Japanese version of the Montreal Cognitive Assessment; MCI, mild cognitive impairment; CS-30, 30-s chair stand. Values are means ± standard division (min–max: range). ^a^ Independent *t* test; ^b^ Mann–Whitney U test; ^c^ χ^2^ test. vs. women. * *p* < 0.05.

**Table 2:** Characteristics of participants with and without mild cognitive impairment (MCI).

	Women				Men			
	non-MCI	MCI	*p*-Value		non-MCI	MCI	*p*-Value	
	(n = 73)	(n = 101)			(n = 28)	(n = 69)		
Age (years)	83.9 ± 4.9	85.6 ± 4.5	0.013	^b^*	85.9 ± 5.0	86.2 ± 4.1	0.528	^b^
Height (cm)	150.2 ± 4.9	149.5 ± 6.0	0.408	^a^	162.1 ± 5.1	163.7 ± 5.5	0.169	^a^
Weight (kg)	47.7 ± 7.0	48.4 ± 8.0	0.513	^a^	60.1 ± 7.1	59.9 ± 7.3	0.771	^b^
BMI (kg/m^2^)	21.2 ± 3.0	21.7 ± 3.6	0.352	^a^	23.0 ± 2.2	22.4 ± 2.5	0.239	^a^
SMI (kg/m^2^)	5.64 ± 0.57	5.53 ± 0.67	0.454	^b^	7.21 ± 0.78	6.95 ± 0.72	0.123	^a^
MoCA-J score (points)	27.4 ± 1.2	21.7 ± 3.0	<0.001	^b^*	27.2 ± 1.3	22.3 ± 2.9	<0.001	^b^*

Abbreviations: BMI, body mass index; SMI, skeletal muscle index; MoCA-J, the Japanese version of the Montreal Cognitive Assessment; MCI, mild cognitive impairment. Values are means ± standard division. ^a^ Independent *t* test; ^b^ Mann–Whitney U test. * *p* < 0.05.

**Table 3:** Univariate and age-adjusted logistic regression analysis predicting cognitive impairment (MCI) in women.

		n (%)		*p*-Value ^a^		Univariate				Age-Adjusted			
		non-MCI (n = 73)	MCI (n = 101)			OR	95% CI	*p*-Value		OR	95% CI	*p*-Value	
Age				0.053									
	≤84 years (Ref.)	39 (53.4%)	39 (38.6%)			1.00	-	-					
	≥85 years	34 (46.6%)	62 (61.4%)			1.82	(0.99–3.36)	0.054					
Hand grip strength				0.117				0.120				0.362	
	High (Ref.)	30 (41.1%)	28 (27.7%)			1.00	-	-		1.00	-	-	
	Middle	24 (32.9%)	34 (33.7%)			1.52	(0.73–3.16)	0.265		1.32	(0.61–2.85)	0.480	
	Low	19 (26.0%)	39 (38.6%)			2.20	(1.04–4.67)	0.040	*	1.81	(0.80–4.09)	0.154	
Normal gait speed				0.206				0.209				0.395	
	High (Ref.)	28 (38.4%)	30 (29.7%)			1.00	-	-		1.00	-	-	
	Middle	26 (35.6%)	32 (31.7%)			1.15	(0.55–2.38)	0.710		1.05	(0.50–2.20)	0.907	
	Low	19 (26.0%)	39 (38.6%)			1.92	(0.90–4.07)	0.090		1.64	(0.75–3.57)	0.217	
Maximum gait speed				0.030	*			0.033	*			0.098	
	High (Ref.)	31 (42.5%)	27 (26.7%)			1.00	-	-		1.00	-	-	
	Middle	25 (34.2%)	33 (32.7%)			1.52	(0.73–3.15)	0.266		1.41	(0.67–2.97)	0.364	
	Low	17 (23.3%)	41 (40.6%)			2.77	(1.29–5.95)	0.009	*	2.40	(1.08–5.45)	0.032	*
CS-30				0.003	*			0.003	*			0.011	*
	High (Ref.)	32 (43.8%)	23 (22.8%)			1.00	-	-		1.00	-	-	
	Middle	24 (32.9%)	32 (31.7%)			1.86	(0.87–3.93)	0.108		1.79	(0.84–3.81)	0.134	
	Low	17 (23.3%)	46 (45.5%)			3.77	(1.74–8.15)	0.001	*	3.39	(1.53–7.49)	0.003	*

Abbreviations: MCI, mild cognitive impairment; CS-30, 30 s chair stand; OR, odds ratio; CI, confidence interval; Ref., reference. The explanatory variable data were divided into tertiles: high, middle, and low. ^a^ χ^2^ test. * *p* < 0.05.

**Table 4:** Univariate and age-adjusted logistic regression analysis predicting cognitive impairment (MCI) in men.

		n (%)		*p*-Value ^a^	Univariate				Age-Adjusted		
		Non-MCI (n = 28)	MCI (n = 69)		OR	95% CI	*p*-Value		OR	95% CI	*p*-Value
Age				0.210							
	≤84 years (Ref.)	14 (50.0%)	25 (36.2%)		1.00	-					
	≥85 years	14 (50.0%)	44 (63.8%)		1.76	(0.72–4.28)	0.212				
Hand grip strength				0.189			0.200				0.243
	High (Ref.)	12 (42.9%)	19 (27.5%)		1.00	-	-		1.00	-	-
	Middle	10 (35.7%)	23 (33.3%)		1.45	(0.52–4.09)	0.480		1.22	(0.41–3.63)	0.722
	Low	6 (21.4%)	27 (39.1%)		2.84	(0.91–8.91)	0.073		2.61	(0.82–8.30)	0.104
Normal gait speed				0.189			0.200				0.308
	High (Ref.)	12 (42.9%)	19 (27.5%)		1.00	-	-		1.00	-	-
	Middle	10 (35.7%)	23 (33.3%)		1.45	(0.52–4.09)	0.480		1.46	(0.52–4.12)	0.479
	Low	6 (21.4%)	27 (39.1%)		2.84	(0.91–8.91)	0.073		2.53	(0.77–8.25)	0.125
Maximum gait speed				0.097			0.111				0.158
	High (Ref.)	12 (42.9%)	20 (29.0%)		1.00	-	-		1.00	-	-
	Middle	11 (39.1%)	21 (30.4%)		1.15	(0.41–3.18)	0.795		1.06	(0.38–3.01)	0.907
	Low	5 (17.9%)	28 (40.6%)		3.36	(1.02–11.05)	0.046	*	3.01	(0.89–10.19)	0.076
CS-30				0.346			0.352				0.453
	High (Ref.)	11 (39.3%)	17 (24.6%)		1.00	-	-		1.00	-	-
	Middle	7 (25.0%)	23 (33.3%)		2.13	(0.68–6.62)	0.193		1.98	(0.63–6.24)	0.245
	Low	10 (35.7%)	29 (42.0%)		1.88	(0.66–5.34)	0.238		1.71	(0.59–4.96)	0.321

Abbreviations: MCI, mild cognitive impairment; CS-30, 30 s chair stand; OR, odds ratio; CI, confidence interval; Ref., reference. The explanatory variable data were divided into tertiles: high, middle, and low. ^a^ χ^2^ test. * *p* < 0.05.

## Data Availability

The data that support the findings of this study are available from the corresponding author upon reasonable request.
